# Cost Effectiveness of Two Lifestyle Interventions in the Vermont WISEWOMAN Program

**DOI:** 10.5888/pcd16.180417

**Published:** 2019-03-21

**Authors:** Ali Johnson, Matt Maiberger, Siobhan Donegan, Nancy C. Kaplan, Patrick Kinner

**Affiliations:** 1 Vermont Department of Health, Burlington, Vermont

## Abstract

**Introduction:**

Low-income women are disproportionately overweight or obese. The Vermont WISEWOMAN (Well Integrated Screening and Evaluation for Women Across the Nation) program, which serves Vermont women whose annual income is less than 250% of the federal poverty level, pays for members to attend 1 of 2 different evidence-based weight loss programs, Weight Watchers or Curves Complete.

**Purpose and Objectives:**

We evaluated cost effectiveness of the weight-loss programs, conducted from April 2014 through March 2016, to determine which represented the best investment of WISEWOMAN program funds.

**Intervention Approach:**

Vermont WISEWOMAN members who were overweight or obese during screening and who identified weight loss as a goal were invited to participate in 1 of the 2 programs on the basis of their place of residence and local Weight Watchers or Curves Complete contractual agreements with the Vermont WISEWOMAN program.

**Evaluation Methods:**

Program costs and benefits were collected for a 2-year period and used to calculate the cost per participant who completed the program and the cost per participant who achieved the weight reduction goal of a 5% or more loss in body weight.

**Results:**

The cost per participant achieving the weight reduction goal with Curves Complete ($8,613) was approximately 5 times the cost for Weight Watchers ($1,610).

**Implications for Public Health:**

Weight Watchers, the evidence-based program with the simplest administrative structure, was significantly more cost effective than Curves Complete. Results suggest that overweight or obese low-income women aged 30 to 64 can lose 5% or more of their body weight more cost effectively through Weight Watchers than through Curves Complete.

**Summary**

**What is already known about this topic?**

The WISEWOMAN program supports the use of 2 evidence-based weight loss programs: Curves Complete and Weight Watchers. These programs have been proven successful in achieving weight loss in the target audience and were approved interventions by the Centers for Disease Control and Prevention’s Well Integrated Screening and Evaluation for Women Across the Nation (WISEWOMAN) program.

**What is added by this report?**

From 2014 through 2016, Vermont implemented these 2 programs through the WISEWOMAN program. Although both Curves Complete and Weight Watchers are evidence-based for outcomes, there is no research that indicates which is more cost effective.

**What are the implications for public health practice?**

By understanding which weight loss program is more cost effective, entities wishing to support weight loss for health outcomes can make informed decisions about how limited resources are expended.

## Introduction

The rise of obesity in America is well documented: 70.7% of women in the United States are overweight or obese (1,2). This number has grown steadily in the past 15 years (3) and is particularly true for women with incomes at or below 130% of the federal poverty level (FPL), for whom the obesity rate is 42%, compared with 29% for women with incomes at or above 350% of the FPL (4). Along with smoking, inadequate exercise and poor nutrition are responsible for 4 chronic diseases (cancer, cardiovascular disease, diabetes, and lung disease) that lead to more than 50% of deaths in the United States (5) and more than 75% of health care expenditures (6).

A 5% weight loss is associated with a 50% reduction in the incidence of type 2 diabetes (7). By reducing weight by 5% to 10%, a woman can improve her glycemic measures, triglycerides, blood pressure, and high-density lipoprotein cholesterol (8), thereby lowering cardiac risk factors (9,10). In people who are obese (have a body mass index [BMI, weight in kilograms divided by height in meters squared] of ≥30), a 5% reduction in body weight is associated with a significant decrease in medical expenditures (11).

Despite the health risks associated with excess body weight, research shows that the behavior change needed to curb overweight and obesity is difficult for many people (12–14). Many try evidence-based weight loss interventions such as Weight Watchers or Curves Complete when they want help with losing weight (15). These interventions are effective at helping people lose weight (15,16) but can be expensive. Reducing the costs of participation in these programs may help low-income women use them. Because these evidence-based interventions are for profit, and because low-income women are not generally part of their target market, few efforts have been made to offer these interventions to this population. Weight Watchers research from the Tennessee Medicaid program (17) and subsidized Weight Watchers programming in Wisconsin, Pennsylvania, New York, and Arizona (18) are examples of effort to reach low-income people with these programs. Health departments partnered with Weight Watchers to offer discounted memberships to overweight adults who received assistance from a local, state, or federally subsidized program. The Tennessee Medicaid partnership with Weight Watchers resulted in 20% of enrolled participants losing a clinically significant amount of weight (17). Beyond these findings, little economic data are available on the value of these interventions for low-income women. Weight Watchers has been found to be more cost effective than other weight loss supports with different population types (19,20).

WISEWOMAN (Well Integrated Screening and Evaluation for Women Across the Nation) is funded by the Centers for Disease Control and Prevention (CDC) and provides heart health screening and lifestyle programs for low-income women (21). Vermont WISEWOMAN pays the full cost of participation for members to attend 1 of 2 evidence-based weight loss programs, Weight Watchers or Curves Complete, and administers participation in the programs. WISEWOMAN recruits members statewide, regardless of whether they have a regular primary care provider, in contrast with other states participating in WISEWOMAN, which recruit members through health care providers. Because WISEWOMAN relies on federal funding, it must be efficient in its spending. No evidence exists in the literature to suggest which evidence-based weight loss intervention is more cost effective. Therefore, our study attempted to determine this by analyzing participation in Weight Watchers and Curves Complete under the WISEWOMAN program from April 2014 through March 2016.

Agencies funded through CDC’s WISEWOMAN are required to conduct process and outcome evaluation of their program efforts, including evaluation of their lifestyle programs (22). We chose to evaluate cost effectiveness of our 2 programs by using an economic evaluation method taught through the Prevention Research Center at Washington University in St Louis, Evidence-Based Public Health: A Course in Chronic Disease Prevention (23), and described in *Evidence-Based Public Health* (24).

## Purpose and Objectives

**Figure 1:**
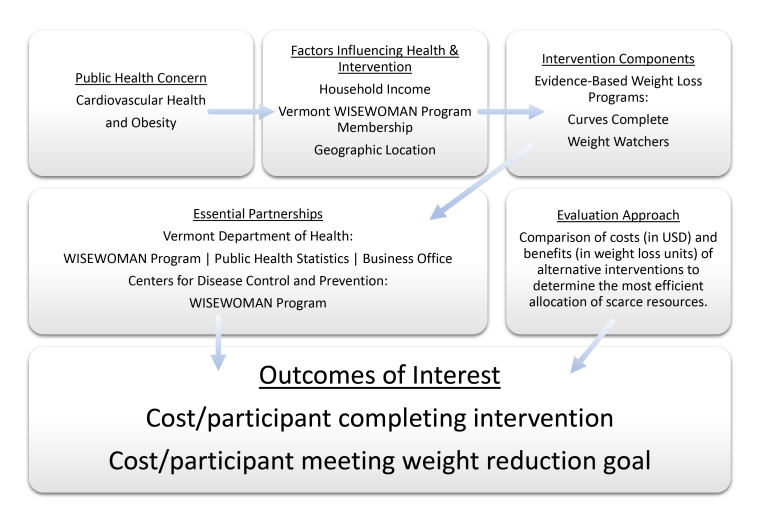
The Vermont WISEWOMAN program and connections among its various components. The program’s objective was improvement of cardiovascular health through weight loss. Abbreviation: WISEWOMAN, Well Integrated Screening and Evaluation for Women Across the Nation.

The goal of our evaluation was to determine which of Vermont WISEWOMAN’s weight loss programs, Weight Watchers or Curves Complete, yielded the most cost effective weight loss by demonstrating the connections among program components, beginning with the public health concern that initiated the program and considering contextual factors, intervention components, partnerships with other organizations, our evaluation approach, and outcomes of interest (Figure 1).

## Intervention Approach

Participants in Vermont WISEWOMAN are routinely screened for heart health risk factors, including measuring height, weight, and blood pressure. To be eligible for these paid screening services, Vermont women need to be aged 30 or older, live in households earning 250% or less of the FPL, and not be enrolled in Medicare Part B. If a participant has a body mass index in the overweight (≥25) or obese (≥30) categories and has identified weight loss as a goal, the WISEWOMAN lifestyle program coordinator invites the member to participate in Weight Watchers or Curves Complete.

Weight Watchers is a group-based social support program (25), and people can join at any time. Weekly 1-hour meetings take place at various community venues and at various times to provide flexibility for participants. The meeting consists of weigh-ins (participant is weighed to track weight-loss progress), social interaction, a presentation by a trained leader, and discussion. Weight Watchers has been operating for more than 40 years. The program format is updated as new scientific information becomes available (17,26,27).

Curves Complete consists of regular fitness workouts each week plus 1 weekly session with a Curves Complete coach (28). The Curves Complete 30-minute exercise circuit works every major muscle group with strength training, cardio exercise, and stretching. The weight management plan consists of the Curves Complete fitness program, a customizable meal plan, and one-on-one coaching and support. The program is based on extensive research (16,29–31) and has been shown to reduce fat, increase lean muscle, boost metabolism, and help women lose weight and maintain weight loss.

During our study (April 2014–March 2016), 350 screened members were found to have a BMI of 25 or greater. Of those members, 274 (78%) were referred to healthy behavior support services (ie, health coaching, lifestyle programs, and community resources for weight loss) by their health care providers and were consequently invited to enroll in Weight Watchers or Curves Complete if they felt motivated and ready to lose weight. Of these 274 women, 56 (20%) chose to enroll. One woman was excluded because she participated in both the Weight Watchers and Curves Complete programs.

**Figure 2:**
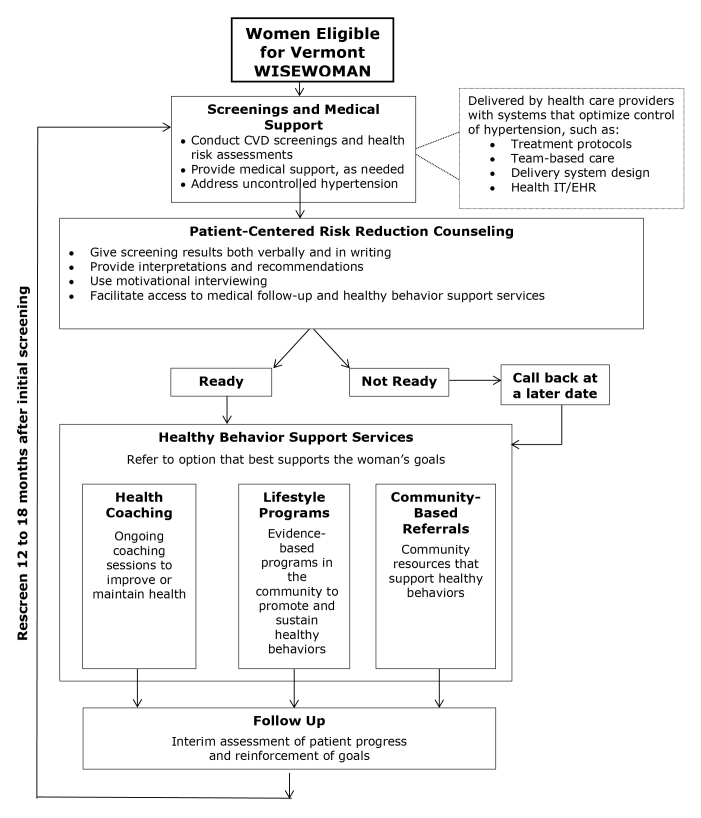
Flow of service delivery for Vermont WISEWOMAN illustrating the process by which eligible women moved from initial screening through the lifestyle program to follow-up. The flow may vary depending on the program structure. Abbreviations: CVD, cardiovascular disease; IT/EHR, information technology/electronic health record; WISEWOMAN, Well Integrated Screening and Evaluation for Women Across the Nation.

In Vermont WISEWOMAN’s service delivery flow for the program (Figure 2), our intervention focused on lifestyle programs in healthy behavior support options. The expected outcomes were improved health behaviors and reduced risk of cardiovascular disease (22).

Criteria for acceptable lifestyle programs were evidence that the proposed program would result in improvement in a woman’s health status by increased physical activity, improved healthy eating, controlled hypertension, weight loss when appropriate, and smoking cessation (22). Members were advised on the types of lifestyle programs available in their geographic area that had contractual agreements with Vermont WISEWOMAN. A postprogram screening was conducted 4 to 6 weeks after completion of the lifestyle program.

## Evaluation Methods

### Data sources

Vermont WISEWOMAN collects personal health information related to risk factor screening, diagnosis, and treatment of heart disease. Patient data are collected on paper forms, and copies of medical records are sent to the Vermont WISEWOMAN program and entered into the Med-IT Data Management System (OxBow Data Management Systems, LLC). The Vermont Department of Health has a public health exemption to collect data on program participants under the Health Insurance Portability and Accountability Act of 1996, and participants consent to having their data used for research, subject to legal restrictions. Our study was determined to fall outside the purview of the Vermont Agency of Human Services institutional review board and did not require its approval or oversight. Lifestyle program costs were collected by the Vermont WISEWOMAN program and captured in the Med-IT Data Management System. Personnel costs were obtained from the Vermont Department of Health Business Office.

### Intervention arms

The criteria for inclusion in our study were 1) membership in Vermont WISEWOMAN, completion of a heart health screening, and a BMI at or above 25, which is considered overweight or obese, before the first session date with Weight Watchers or Curves Complete; and 2) participating in a lifestyle program session date from April 1, 2014, through March 31, 2016, and being in a readiness-to-change stage of preparation, action, or maintenance based on Prochaska and DiClemente’s transtheoretical model of change (32). Women participating in both Curves Complete and Weight Watchers were excluded. This left a final sample size of 56 participants for analysis (17 from Curves Complete and 39 from Weight Watchers).

Participants self-selected 1 of the 2 main intervention arms, Weight Watchers or Curves Complete, based on their place of residence and on local Weight Watchers or Curves Complete contractual agreements with Vermont WISEWOMAN. Weight Watchers classes were offered throughout the state, and Curves Complete sessions were available in 5 communities. The Weight Watchers intervention consisted of at least 12 meetings in 24 weeks. Weight Watchers participants could further opt to partake in fitness activities, defined as specific community resources, such as YMCA day passes, Jazzercise, pool memberships, fitness classes, and other gym memberships. Therefore, Weight Watchers participants were grouped as follows: Weight Watchers (total), Weight Watchers without fitness, and Weight Watchers with fitness. The Curves Complete intervention consisted of at least 10 sessions in 12 weeks. Curves Complete membership consisted of circuit training, fitness classes, weight-loss tracking, one-on-one health coaching, and menu planning.

### Definitions of variables 

Intervention time was calculated at program completion as the number of weeks between the first and last session (or last session attended if incomplete, or last session attended before a hiatus from sessions of 1 month or longer for intermittent participants). Benefits were defined as 1) the percentage of participants who completed the lifestyle program and 2) the percentage of participants who lost 5% or more of their body weight. Participants were considered to have completed a program if they attended the required number of sessions in the allotted time. Women who participated intermittently (had more than 1 month between sessions) were classified as incomplete.

Weight loss was calculated as the participant’s end weight minus start weight. Start weight was the participant’s weight measured at the first session of Weight Watchers or Curves Complete attended. End weight was the participant’s weight measured at the last session during which they completed the intervention (or last session attended if incomplete or last session attended before 1 month or longer hiatus from sessions for intermittent participants). The percentage of body weight lost was calculated as the participant’s weight loss divided by her start weight and multiplied by 100. BMI was the participant’s body weight in kilograms (measured by a Weight Watchers or Curves Complete staff member at a session) divided by her height in meters squared (measured by a health care provider at a routine screening). A BMI of more than 18.5 and less than 25.0 indicated a normal or healthy weight (33).

We considered program costs to determine which of the 2 programs was more cost effective. Costs were divided into 2 main categories: lifestyle program costs and personnel costs. Lifestyle program costs included membership fees and incentives attributable to the Weight Watchers and Curves Complete programs and were generally calculated on a participant-by-participant basis. They also included membership fees and incentives for the Weight Watchers fitness component, which was paid for a subset of the Weight Watchers group and which occurred concurrently with the weight-loss program (8 women participated in the fitness component). Personnel costs were labor (salary) and fringe costs (insurance, leave accrual, and other benefit) of WISEWOMAN program staff members that were attributable to Weight Watchers and Curves Complete program administration.

Membership costs were intervention-specific. Weight Watchers membership costs for each participant were calculated as the number of vouchers (for admission to Weight Watchers sessions) the participant used during the study period multiplied by the cost per voucher. For Weight Watchers participants using a program-paid fitness membership, membership fees and incentives costs were included in the lifestyle program costs.

The Curves Complete cost for each participant was the 3-month membership fee invoiced for each participant, which was dependent on the Curves Complete location. Curves Complete membership costs were incurred at the beginning of a participant’s program, regardless of level of use.

Incentives costs were intervention-specific and participant-specific. Weight Watchers participants received a points calculator for attending 6 sessions and a cookbook for completing the program. Curves Complete participants and Weight Watchers participants concurrently using a program-paid fitness membership received a sports brassiere for beginning the intervention and a gift card for a sporting goods store for completing the intervention. Participants receiving incentives were identified, and the associated cost of each incentive was included. Sunk costs, such as current inventory of cookbooks and points calculators, were excluded. The study focused on costs directly attributable to the current study population rather than potential future participants. Sunk incentive costs were negligible relative to personnel costs.

We included all lifestyle program costs incurred for eligible participants between the first lifestyle program session and the session at which end weight was measured, regardless of whether the woman completed the program before the study cut-off date of March 2016. For example, if a participant had a first lifestyle program date in March 2016 and completed the intervention in June 2016 (after the study cut-off date for first lifestyle program date), all program costs incurred during this timeframe for this participant would be included. All program costs associated with eligible participants were included, regardless of whether participants completed the program. For participants who completed the full number of lifestyle program sessions but in a longer amount of time than allotted (24 weeks for Weight Watchers, 12 weeks for Curves Complete), only costs incurred within the allowed time were included. For participants who participated intermittently, only the cost of the first set of consecutively attended sessions were included.

Personnel costs consisted of salary, fringe benefits, and paid leave (34) incurred from April 1, 2014, through March 31, 2016. We obtained these costs from quarterly Vermont Department of Health Business Office reports of expenses billed to the WISEWOMAN cost center. 

Each employee’s salary cost was computed as the total actual salary (in USD) billed to WISEWOMAN, multiplied by the estimated percentage of the time the employee spent on Weight Watchers and Curves Complete, multiplied by the percentage of time spent on administration. To calculate personnel expenses attributable to each of the 2 lifestyle programs, we took the total salary billed for each employee to the WISEWOMAN program, multiplied that by the estimated percentage of that employee’s time spent on Weight Watchers or Curves Complete. Cost of each employee’s fringe benefits attributable to Weight Watchers and Curves Complete was computed by the same method as salary cost by using total fringe benefits in US dollars billed to WISEWOMAN. All employee salary and fringe benefits costs for all quarters were totaled and tabulated by intervention type. Small salary costs, under 0.05 full time equivalent, were excluded because they were difficult to collect consistently for the 2-year study period and were likely to have a negligible effect.

### Statistical analyses

We used Excel 2016 (Microsoft Corp) to calculate 3 types of cost effectiveness measures as cost per success (completed intervention or met weight reduction goal). Each cost effectiveness ratio was calculated as the total cost to WISEWOMAN divided by the total benefit, that is, the average cost per participant divided by the percentage of participants with a success. For example, the cost per participant completing the intervention was the sum of the program and personnel costs attributable to an intervention divided by the number of participants who completed that intervention. The denominator was changed to the number of participants who met the weight reduction goal to calculate other cost effectiveness ratios.

We used SAS 9.3 (SAS Institute Inc) to test significance for each benefit and population characteristic. To generate 95% confidence limits for sample proportions, we used Wilson score approximation, which has been shown to perform well with small sample sizes (35). A difference was considered significant if the confidence intervals of the 2 lifestyle programs being compared did not overlap.

Because personnel costs were expected to be a substantial driver of costs, a sensitivity analysis was conducted to vary key assumptions used to compute those costs and to explore how variability in these assumptions affected the comparison of cost effectiveness ratios for the lifestyle program. We attempted to model the variability of personnel costs that could be encountered by other WISEWOMAN programs in other locations and at different phases of implementation. We explored 3 scenarios: 1) to simulate equal program administration costs for both intervention arms, personnel costs attributed to Curves Complete and Weight Watchers were assumed to be equal; 2) to simulate a more labor-intensive start-up phase, total staff time attributable to the administration of both lifestyle programs was increased by 5%; and 3) to simulate a less labor-intensive maintenance phase, total staff time attributable to the administration of both lifestyle programs was decreased by 5%.

## Results

Of the 56 participants included in the study, 39 were in Weight Watchers and 17 in Curves Complete (Table 1). Of the 56 participants, 24 had a choice based on geographic location to attend either Weight Watchers or Curves Complete, whereas 32 could only attend Weight Watchers because Curves Complete was not available nearby. Of the 24 women who lived near a Curves Complete franchise and could choose either Curves Complete or Weight Watchers, 16 chose to attend Curves Complete. Of the 39 participants in the Weight Watchers intervention arm, 31 opted for Weight Watchers without fitness and 8 selected Weight Watchers with fitness.

**Table 1:** Characteristics of Study Population (N = 56) by Intervention Arm, Study of Cost Effectiveness of Two Lifestyle Interventions in the Vermont WISEWOMAN Program, April 2014–March 2016^a^

Characteristic	Total (N = 56)	Curves Complete (n = 17)	Weight Watchers (n = 39)
**Body mass index^b^ at office visit before starting lifestyle program**			
25.0–29.9 (Overweight)	14	6	8
30.0–34.9 (Obese class I)	15	2	13
35.0–39.9 (Obese class II)	14	4	10
≥40.0 (Obese class III)	13	5	8
**Readiness to change^c^**			
Preparation	17	7	10
Action	38	10	28
Maintenance	1	0	1
**Age at first session, y**			
30–39	10	3	7
40–49	15	4	11
50–59	21	7	14
≥60	10	3	7
**Race/ethnicity**			
White, non-Hispanic	50	13	37
Other race	6	4	2

Abbreviation: WISEWOMAN, Well Integrated Screening and Evaluation for Women Across the Nation.

a Values are number.

b Weight in kilograms divided by height in meters squared.

c Based on Prochaska and DiClemente’s transtheoretical model of change (32).

Participants’ BMI values before enrollment were relatively evenly distributed among the 4 BMI categories: 14 participants were overweight (BMI 25.0–29.9), 15 were obese class I (BMI 30.0–34.9), 14 were obese class II (BMI 35.0–39.9), and 13 were obese class III (BMI ≥40.0). Thirty-eight participants were assessed to be at the Action level in their readiness to change assessments, 17 at the Preparation phase, and 1 at the Maintenance phase. The age of participants ranged from 31 to 75 years. Twenty-one participants were aged 50 to 59, followed by 15 aged 40 to 49, 10 aged 30 to 39, and 10 aged 60 or older. Fifty participants were non-Hispanic white, and 6 identified race/ethnicity as something other than non-Hispanic white. No significant differences were observed between the Curves Complete and Weight Watchers study groups before enrollment with respect to BMI, readiness to change, age, and race/ethnicity.

The mean intervention time for all Curves Complete participants was 7.8 weeks, 9.6 weeks for those completing the intervention and 10.0 weeks for those meeting the weight reduction goal. The mean intervention time for all Weight Watchers participants was 9.9 weeks, 13.6 weeks for those completing the intervention, and 12.5 weeks for those meeting the weight reduction goal. Ten Curves Complete participants and 20 Weight Watchers participants completed the lifestyle program. Four Curves Complete participants and 17 Weight Watchers participants met the weight reduction goal. The different rates of completion and goal achievement for the 2 programs were not significant.

**Figure 3:**
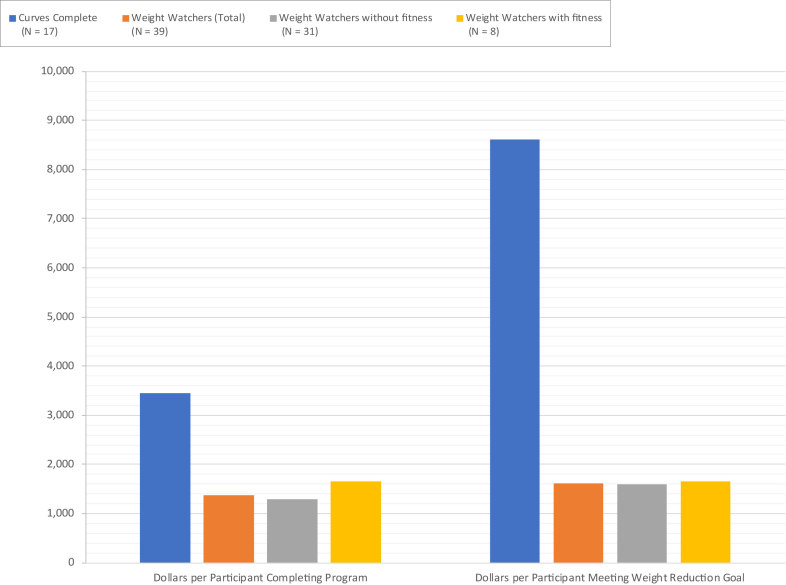
Cost effectiveness of lifestyle programs by intervention: Curves Complete**,** Weight Watchers (total), Weight Watchers without fitness, and Weight Watchers with fitness, Vermont WISEWOMAN Program, April 2014–March 2016.

The total cost was $34,453 for the 17 Curves Complete participants and $27,374 for the 39 Weight Watchers participants (Table 2). Personnel costs accounted for 87% of the total Curves Complete cost and 79% of the total Weight Watchers cost. The average per-participant cost was $2,027 for Curves Complete and $702 for Weight Watchers. The cost per participant completing the intervention with Curves Complete ($3,445) was approximately 2.5 times the cost for Weight Watchers ($1,369) (Figure 3). The cost per participant meeting the weight reduction goal with Curves Complete ($8,613) was approximately 5 times the cost for Weight Watchers ($1,610).

**Table 2:** Costs by Intervention Arm, Study of Cost Effectiveness of Two Lifestyle Interventions in the Vermont WISEWOMAN Program, April 2014–March 2016

Cost, $	Curves Complete, n = 17	Weight Watchers, n = 39
**Total**	34,453.10	27,373.85
**Lifestyle program**		
Membership	4,048.50	4,048.00
Incentives	391.50	639.58
Additional fitness	0.00	1,102.79
Subtotal for lifestyle program	4,440.00	5,790.37
**Personnel**		
Labor	22,093.48	15,968.13
Fringe costs^a^	7,919.62	5,615.35
Subtotal for personnel	30,013.10	21,583.48
**Per participant, mean**		
Personnel	1,765.48	553.42
Lifestyle program cost	261.18	148.47
Total per participant	2,026.66	701.89

Abbreviation: WISEWOMAN, Well Integrated Screening and Evaluation for Women Across the Nation.

a Insurance, leave accrual, and other employee benefits.

**Table:** 

Cost Effectiveness Measure	Curves Complete, n = 17	Weight Watchers (Total), n = 39	Weight Watchers Without Fitness, n = 31	Weight Watchers With Fitness, n = 8
Cost per participant completing program, $	3,445	1,369	1,299	1,648
Cost per participant meeting weight-loss goal, $	8,613	1,610	1,599	1,648

To control for the effect of physical activity interventions occurring concurrently with Weight Watchers, we calculated the cost effectiveness ratios for the 2 Weight Watchers subgroups: Weight Watchers without fitness and Weight Watchers with fitness. The cost per participant completing the intervention with Curves Complete ($3,445) was approximately 2.5 times the cost for Weight Watchers without fitness ($1,299) and approximately 2 times the cost for Weight Watchers with fitness ($1,648). The cost per participant meeting the weight-loss goal with Curves Complete ($8,613) was approximately 5 times the cost for either Weight Watchers without fitness ($1,599) or Weight Watchers with fitness ($1,648).

The sensitivity analysis found that varying the personnel costs to model 3 different scenarios (equal personnel costs between Weight Watchers and Curves Complete, an increase of 5% in personnel costs, and a decrease of 5% in personnel costs) still resulted in Curves Complete being less cost effective than Weight Watchers. In all 3 scenarios, the Curves Complete cost per participant completing the intervention was at least 1.5 times the cost of Weight Watchers (total), Weight Watchers without fitness, or Weight Watchers with fitness; in scenarios 2 and 3, the Curves Complete cost per participant completing the program were more than 2 times the Weight Watchers cost. In all 3 scenarios, the Curves Complete cost per participant meeting the weight reduction goal was at least 4 times the cost of Weight Watchers (total), Weight Watchers with fitness, and Weight Watchers without fitness; in scenarios 2 and 3, the Curves Complete cost per participant meeting the weight reduction goal was more than 5 times the Weight Watchers costs.

## Implications for Public Health

Because of the results of our evaluation, Vermont WISEWOMAN now refers members to Weight Watchers whenever possible. Our evaluation showed that Weight Watchers was more cost effective than Curves Complete for both number of women completing the intervention and those achieving a 5% or greater weight loss, and the cost per participant meeting the weight reduction goal with Curves Complete was approximately 5 times the cost for Weight Watchers. Also, Weight Watchers had a simpler administrative structure — mailed vouchers and single point of contact — than Curves Complete, which required invoiced payment for classes, communication with multiple local franchises, and collaboration with the national organization. These results are supported by similar studies that used different designs and populations (19,20). Study results may be of value to any public health organization that partners with multiple evidence-based weight loss interventions, especially Weight Watchers or Curves Complete.

Our study had several limitations. These were primarily due to the small, rural nature of the geographic area, the specific population studied, and the difficulty of evaluating personnel costs associated with any fitness participation in the Weight Watchers intervention. Approximately 61% of Vermont women live in rural areas (36). Therefore, our findings may be most applicable to programs directed at small, rural populations where weight loss among low-income women is the objective; results may not be generalizable to urban areas. Our participants’ demographics and our focus on low-income women yielded a small population from which to draw and a small number of study participants. Approximately 151,200 women aged 30 to 65 live in Vermont (37). Only 56 participated in the study, and 30 completed an intervention, although we recruited participants for 2 years to try to increase the numbers. Selection bias could have occurred because each participant self-selected an intervention rather than being randomly assigned. Women could have characteristics that lead them to choose one intervention over another that we did not adjust for in our analysis.

Cost analysis also could have been a study limitation because of the grouping of personnel costs related to establishing partnerships and to participation phases for Curves Complete and Weight Watchers. These costs were combined because the study took place from the initiation of the 2 interventions through 2 years of enrollment and participation. If one intervention required a disproportionately higher number of personnel hours to establish than the other, total costs could have been overestimated and cost effectiveness underestimated. Therefore, caution should be used in comparing these results to interventions that are exclusively in the active participation phase. Nevertheless, our study results may be useful to public health organizations that partner with multiple evidence-based weight loss interventions such as Weight Watchers or Curves Complete, where administration costs factor into analysis of cost effectiveness.

The most significant limitation of our study is that Weight Watchers costs excluded personnel costs attributable to administration of its fitness program. Participants could engage in self-directed physical activity unknown to us and participate in program-referred services promoting physical activity. Eight Weight Watchers participants used a fitness membership concurrently. We were unable to compute the personnel costs for administering such memberships; therefore, those costs were omitted, although the costs for the fitness memberships were included. We tried to account for this limitation by reporting Weight Watchers cost effectiveness ratios in subgroups (with fitness, without fitness, and total) and by conducting a sensitivity analysis modeling equal personnel costs for the 2 interventions. Future studies could include an intention-to-treat analysis for each intervention, regardless of length of participation, considering the cost per unit of effect, because such an analysis could demonstrate actual cost effectiveness.

Although Weight Watchers venues were evenly distributed across the state, Curves Complete venues were not. Bias could have been introduced if this geographic factor influenced intervention completion or weight loss attainment. Women enrolling in either program were encouraged to select the option that they perceived as having the best overall fit and to consider the proximity of the program to their home, and any prior knowledge they had of the program. We had no expectation or evidence that women enrolling in Curves Complete would be more or less successful than those enrolling in Weight Watchers.

Weight Watchers could be more biased toward goal achievement than Curves Complete, because it allots more time to achieve the 5% weight loss. Curves Complete participants had 12 weeks to achieve the goal (with an intensive program), and Weight Watchers participants had 24 weeks (with a less intensive program). This bias was likely mitigated because Weight Watchers participants who met the weight reduction goal used a smaller proportion of their allotted time than did Curves Complete participants. The mean percentage of the allotted time for the interventions for participants who met the weight reduction goal was 83% for Curves Complete and 52% for Weight Watchers. We were unable to determine whether weight loss was maintained. Research indicates that maintaining weight loss is difficult for many people (12), and no compelling evidence is available to show whether Weight Watchers or Curves Complete is successful at aiding in weight loss maintenance.

Intervention outcomes may have been affected by characteristics of WISEWOMAN participants, which would potentially decrease the generalizability of the results in interventions that serve a more diverse population or that use a clinic-based (rather than statewide) recruitment model. Also, information from our study may be less valuable in a program where participants are expected to pay for their own membership or class fees, there is a third party to cover these expenses, less administrative data are collected, or different incentives are offered.

We described a method to assess cost effectiveness of weight loss interventions for low-income women administered by a state public health department. Because excess weight is a risk factor for chronic diseases and because disparities exist among socioeconomic groups, our evaluation can help determine the most cost effective approach to administering lifestyle programs among low-income women.
